# Thymomectomy versus complete thymectomy in early-stage non-myasthenic thymomas: a multicentric propensity score-matched study

**DOI:** 10.1093/icvts/ivac167

**Published:** 2022-06-20

**Authors:** Emanuele Voulaz, Gianluca Perroni, Anna Russo, Alexandro Patirelis, Giuseppe Mangiameli, Marco Alloisio, Vincenzo Ambrogi

**Affiliations:** IRCCS Humanitas Research Hospital, Department of Thoracic Surgery, Milan, Italy; Department of Biomedical Sciences, Humanitas University, Milan, Italy; Division of Thoracic Surgery, Department of Thoracic Surgery, Tor Vergata University Polyclinic, Rome, Italy; Division of Thoracic Surgery, Department of Thoracic Surgery, Tor Vergata University Polyclinic, Rome, Italy; Division of Thoracic Surgery, Department of Thoracic Surgery, Tor Vergata University Polyclinic, Rome, Italy; IRCCS Humanitas Research Hospital, Department of Thoracic Surgery, Milan, Italy; Department of Biomedical Sciences, Humanitas University, Milan, Italy; IRCCS Humanitas Research Hospital, Department of Thoracic Surgery, Milan, Italy; Department of Biomedical Sciences, Humanitas University, Milan, Italy; Division of Thoracic Surgery, Department of Thoracic Surgery, Tor Vergata University Polyclinic, Rome, Italy

**Keywords:** Thymoma, Surgery, Thymomectomy, Complete thymectomy, Myasthenia gravis

## Abstract

**OBJECTIVES:**

Thymomectomy is gaining consensus over complete thymectomy in early-stage thymoma without myasthenia gravis. This is due both to the difficulty of establishing prospective and randomized controlled studies and to the lack of well-defined selection criteria. This bicentric, retrospective propensity score-matched study aims at comparing oncological outcomes, measured in terms of overall survival and thymoma-related survival, in patients undergoing either thymomectomy or complete thymectomy.

**METHODS:**

We retrospectively analysed medical records of patients with clinical early-stage (I and II) thymoma undergoing thymomectomy or complete thymectomy. Exclusion criteria were the presence of myasthenia gravis, clinical advanced tumours and thymic carcinoma. A propensity score-matching analysis was applied to reduce potential preoperative selection biases such as comorbidity (Charlson score), tumour maximal diameter and surgical approach (open versus minimal). All variables were dichotomized.

**RESULTS:**

A total of 255 patients were enrolled from 2 different Hospitals, 126 underwent complete thymectomy and 129 a thymomectomy. Disease-free and thymoma-related survivals showed a 5-year rate of 87.7% and 96.0% and a 10-year rate of 82.2% and 91.9%, respectively. Propensity score-matching analysis selected a total of 176 patients equally divided between the 2 groups. No difference was found for both disease-free (*P* = 0.11) and thymoma-related (*P* = 0.37) survival in the 2 groups of resection. Multivariable Cox regression analysis showed that histology (*P* < 0.001), residual disease (*P* < 0.001) and adjuvant chemotherapy (*P* < 0.001) were the only predictors of shorter disease-free survival. Whereas there was no evidence to confirm that disease-free and thymoma-related survivals were influenced by resection extent.

**CONCLUSIONS:**

Thymomectomy is an adequate surgical resection for non-myasthenic thymoma, achieving disease-free and thymoma-related survivals comparable to those after complete thymectomy.

**Figure:**
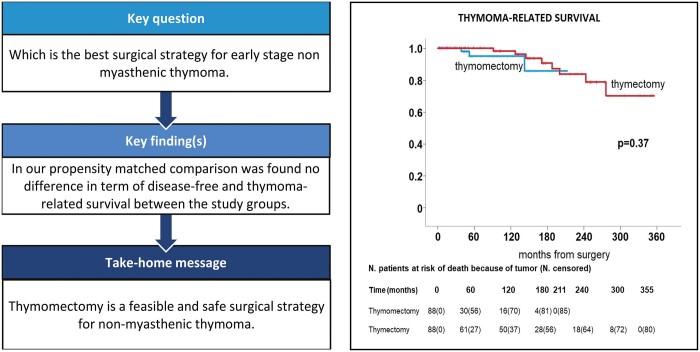

## INTRODUCTION

The extent of thymic resection in case of thymoma is still matter of debate. Complete thymectomy through median sternotomy is considered the standard treatment of thymic epithelial tumours without myasthenia gravis [1]. This procedure consists in a radical surgical resection of the tumour including the entire thymus gland. Yet, in the presence of myasthenia gravis extended thymectomy that also includes the mediastinal fat tissue anterior to the pericardium, aorta, and superior vena cava between the 2 phrenic nerves, from the thyroid gland to the diaphragm, added better neurological control [2, 3].

However, the increasing use of minimally invasive approaches such as video-assisted thoracic surgery (VATS) has progressively evoked limited resections as easily more accomplishable through this route and yet oncologically adequate [3]. In this setting, limited thymectomy, that is the complete resection of thymoma along with surrounding thymus and fatty tissue leaving the healthy thymus gland, proved to be oncologically effective in a single-centre, case–control, retrospective study [4] and propensity score-matching analyses [5]. More recently, thymomectomy (i.e. simple removal of the thymoma) was also introduced with discordant results [1, 3, 6, 7]. A 2016 propensity score analysis for stage I thymoma found a difference in the rate of local recurrence without reaching statistical significance [8], and another 2018 retrospective analysis of stage I-IV by one of us had a comparable 5- and 10-year disease-free survivals [9]. Furthermore, a recent study of a European Society of Thoracic Surgeons Thymic Working Group Study [10] differentiated the surgical resection into thymothymectomy and simple thymomectomy: the former representing a complete resection removing both the tumour and entire thymus tissue and the latter the sole resection of the tumour without thymus gland. They concluded that simple thymomectomy was associated with a shorter progression-free survival compared with thymothymectomy [10].

Due to the rarity of this disease, current evidence is largely based on non-randomized retrospective studies with few patients enrolled. These studies differ by sample size, open surgical approach (sternotomy or thoracotomy), extent of resection and disease stage. Furthermore, very long follow-up times are required to conduct a retrospective analysis since the slow tumour growth.

Our 2 centres gathered a pluridecennial experience on thymectomy for nonmyasthenic thymoma that allows the building of a consistent database. Looking at operative notes, we identified 2 major kinds of operations: one limited to tumour mass excision that we described as thymomectomy and the other including the healthy thymus and upper poles. The aim of this study was to retrospectively analyse a cohort of patients treated for early-stage thymoma without myasthenia gravis by either thymomectomy or complete thymectomy over a very long follow-up period, to better detect the oncological outcomes of this slow-growing neoplasm.

## MATERIALS AND METHODS

We reviewed medical records of 514 patients from 2 major centres devoted to mediastinal surgery who underwent surgical resection of thymoma between 1986 and 2019.

### Ethics statement

All patients signed an informed consent for the acquisition and the usage of clinical data for research purposes at admission. The study was approved by the internal research boards of both centres (Institute Humanitas Clinical & Research Hospital and Tor Vergata University Polyclinic).

### Study design

**Figure 1:**
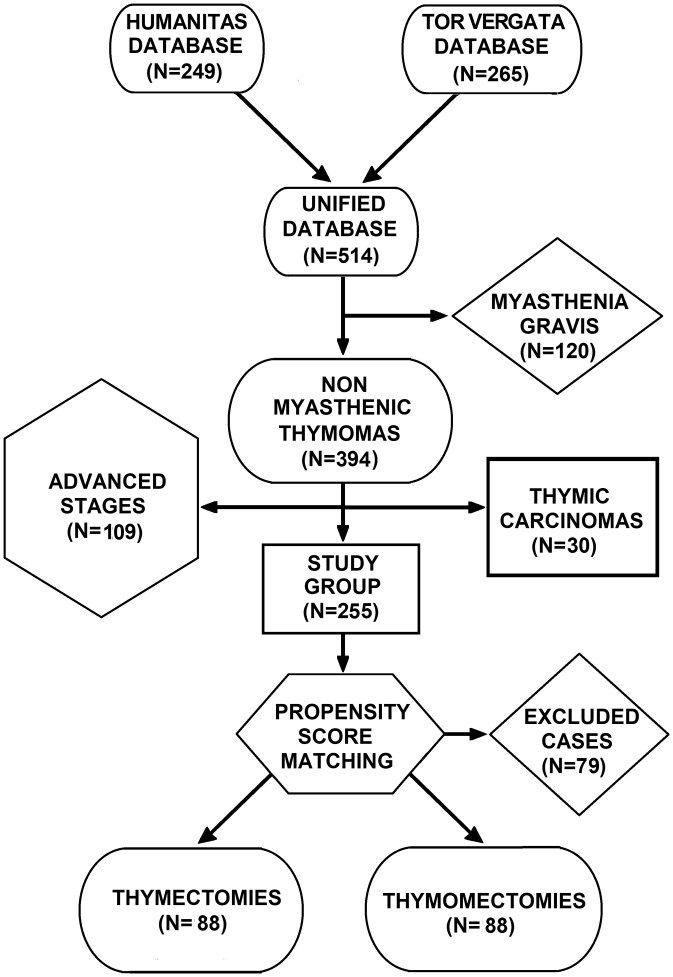
Patient selection flow chart.

This is a retrospective study aimed at evaluating the long-term outcome of thymomectomy compared to complete thymectomy. To reduce the selection bias, the 2 populations were selected and matched one to one by a propensity score-matching analysis. To avoid confounding factors, patients with myasthenia gravis (*n* = 120) and clinical advanced stage, as stage III or more at preoperative staging (*n* = 109), were excluded. Thymic carcinomas (*n* = 30) were also excluded because of different clinical behaviours (Fig. 1). Therefore, the final study group accounted for a total of 255 patients. Demographic data and surgical details are summarized in Table 1.

**Table 1:** Patients’ characteristics according to the kind of surgery before and after propensity score matching

	Before matching				After matching			
	Thymomectomy (*n* = 129)	Thymectomy (*n* = 126)	*P*-Value	Standardized difference	Thymomectomy (*n* = 88)	Thymectomy (*n* = 88)	*P*-Value	Standardized difference
Age >56 years, *n* (%)	78 (60.5)	59 (46.8)	0.029	0.28	59 (67.0)	35 (39.7)	<0.001	0.57
Gender male, *n* (%)	67 (51.9)	76 (60.3)	0.18	0.17	44 (50.0)	52 (59.1)	0.23	0.18
Charlson index, *n*(%)			0.49	0.09			1.00	0.00
0–2	67 (51.9)	60 (47.6)			45 (51.1)	45 (51.1)		
3–7	62 (48.1)	66 (52.4)			43 (48.9)	43 (48.9)		
Stage Masaoka, *n* (%)			0.11	0.20			0.54	0.09
I	65 (50.4)	51 (40.5)			41 (46.6)	37 (42.0)		
II–III	64 (49.6)	75 (59.5)			47 (53.4)	51 (58.0)		
Tumour size >63 mm, *n* (%)	67 (51.9)	61 (48.4)	0.57	0.07	54 (61.4)	54 (61.4)	1.00	0.00
Histology WHO, *n*(%)			0.91	0.01			1.00	0.00
A–AB–B1	100 (77.5)	97 (77.0)			65 (73.9)	65 (73.9)		
B2–B3	29 (22.5)	29 (23.0)			23 (26.1)	23 (26.1)		
Approach, *n* (%)			<0.001	0.49			1.00	0.00
Minimal	46 (35.7)	19 (15.1)			17 (19.3)	17 (19.3)		
Open	83 (64.3)	107 (84.9)			71 (80.7)	71 (80.7)		
Residual, *n* (%)	8 (6.2)	15 (11.9)	0.11	0.20	3 (3.4)	10 (11.4)	0.044	0.31
Chemotherapy, *n* (%)			<0.001	0.91			<0.001	0.92
Adjuvant	9 (7.0)	54 (42.9)			2 (2.3)	31 (35.2)		
None	120 (93.0)	72 (57.1)			86 (97.7)	57 (64.8)		
Radiotherapy, *n* (%)			0.79	0.03			0.14	0.23
Adjuvant	14 (10.9)	15 (11.9)			6 (6.8)	12 (13.6)		
None	115 (89.1)	111 (88.1)			82 (93.2)	76 (86.4)		
Death related to thymoma, *n* (%)	10 (7.7)	17 (13.5)	0.14	0.19	3 (3.4)	8 (9.1)	0.12	0.24
Deaths, *n* (%)	27 (20.9)	18 (14.3)	0.16	0.17	22 (25.0)	13 (14.8)	0.089	0.26
Follow-up, months, mean ± SD	64.1 ± 51.5	153.6 ± 107.8	<0.001	1.06	59.6 ± 54.8	141.7 ± 103.8	<0.001	0.99
Disease-free interval, months, mean ± SD	60.5 ± 50.4	132.6 ± 110.0	<0.001	0.84	57.9 ± 54.6	122.9 ± 104.8	<0.001	0.78
Time to recurrence, months, mean ± SD	54.3 ± 42.0	32.6 ± 37.9	0.14	0.54	50.7 ± 35.8	26.5 ± 35.2	0.17	0.68
Recurrence, *n* (%)	11 (8.5)	24 (19.0)	0.015	0.31	6 (6.8)	16 (18.2)	0.023	0.35
Recurrence site, *n* (%)			0.95	0.02			0.26	0.53
Local	4 (36.4)	9 (37.5)			3 (50.0)	4 (25.0)		
Distant	7 (63.6)	15 (62.5)			3 (50.0)	12 (75.0)		

SD: standard deviation; WHO: World Health Organization.

Histology was classified according to the latest World Health Organization (WHO) classification system, specimens obtained before 1999 and originally classified according to Muller-Hermelink were transformed into WHO classification according to the currently available conversion table [11]. Staging was performed using the Masaoka-Koga staging system [12–14].

Complete thymectomy was defined as complete removal of the tumour mass *en bloc* with the entire thymus and the upper poles. Conversely, thymomectomy was described as the resection of the encapsulated tumour in clean margins with the surrounding and indissociable thymus gland, leaving the residual thymic and fatty tissue and upper poles. Both sternotomy and thoracotomy were grouped together as ‘open’. The VATS technique was usually carried out in both centres through 3 ports with ‘diamond shape’ disposition. One of the centres introduced robotic surgery (Da Vinci^®^ surgical system) in 2016 using 3 incisions. All these techniques (VATS and robotic surgery) were grouped together as ‘minimal’. Totally, 8 surgeons were involved in the care of the patients of this study. All the patients underwent surgery, both thymomectomy and complete thymectomy, were scheduled to a multidisciplinary meeting with radiologists, oncologists, pathologists and thoracic surgeons and adjuvant therapy was indicated in case of advanced tumour.

### Statistical analysis

Statistical analysis was performed using SPSS (IBM Corp. Released 2016. IBM SPSS Statistics, Version 24.0. Armonk, NY: IBM Corp.). *P*-value <0.05 was considered statistically significant. Descriptive analysis of data with normal distribution was performed using either Pearson’s Chi-square for categorical variables or Student’s *T*-test for continuous one. For propensity score-matching analysis, we used a logistic regression model with the type of resection (thymomectomy versus thymectomy) as dependent variable and 3 preoperative variables potentially influencing the choice as covariates: Charlson comorbidity index, tumour size and surgical approach. To simplify the evaluation, all variables were dichotomized. For continuous variables, distributed in a Gaussian way, the cut-off value was chosen according to the median. Discrete variables were dichotomized according to the clinical significance (worse versus better prognosis). As previously described, the approach was split into open (e.g. sternotomy and thoracotomy) versus minimal (e.g. triportal VATS and robotic). Cases were matched according the closest estimated propensity score using the following algorithm: 1:1 optimal match with a ±0.02 calliper and no replacement. Validity of the analysis was tested by the standardized difference in pre- and post-matching evaluating the value approximating to zero.

Prognostic factors’ evaluation was conducted with survival curves. Thymoma-related and disease-free survival curves were estimated from the date of surgery and using the Kaplan–Meier method with the log-rank test to evaluate any statistically significant difference. Overall survival was not considered due to the extremely longer follow-up in the thymectomy group and thus influenced by the death for other causes. For thymoma-related survival, we included patients who directly died from disease progression as well as for disease clearly induced by therapies for thymoma control (i.e. myelotoxicity, postactinic pulmonary fibrosis). Afterwards, univariable and multivariable Cox regression analyses were performed to adjust for confounding factors. Age (≤56 versus >56 years), gender (male versus female), Charlson index (0–2 versus 3–7), Masaoka stage (I versus II–III), tumour size (≤63 versus >63 mm), WHO histology (A–AB–B1 versus B2–B3), surgical approach (minimal versus open), extent of resection (thymomectomy versus thymectomy), residual tumour (absent versus present) and adjuvant treatment (no versus yes) were set as covariates. We used Cox regression model because we assumed that the hazard of death or recurrence of disease is constant over the time in the 2 groups of patients.

### Data availability statement

All relevant data are included within the article.

## RESULTS

### Clinical cumulative preliminary analysis

As aforementioned, the final number of the patients considered for the study was 255. Table 1 summarizes major features of this subset accounting 126 thymectomies and 129 thymomectomies.

**Figure 2:**
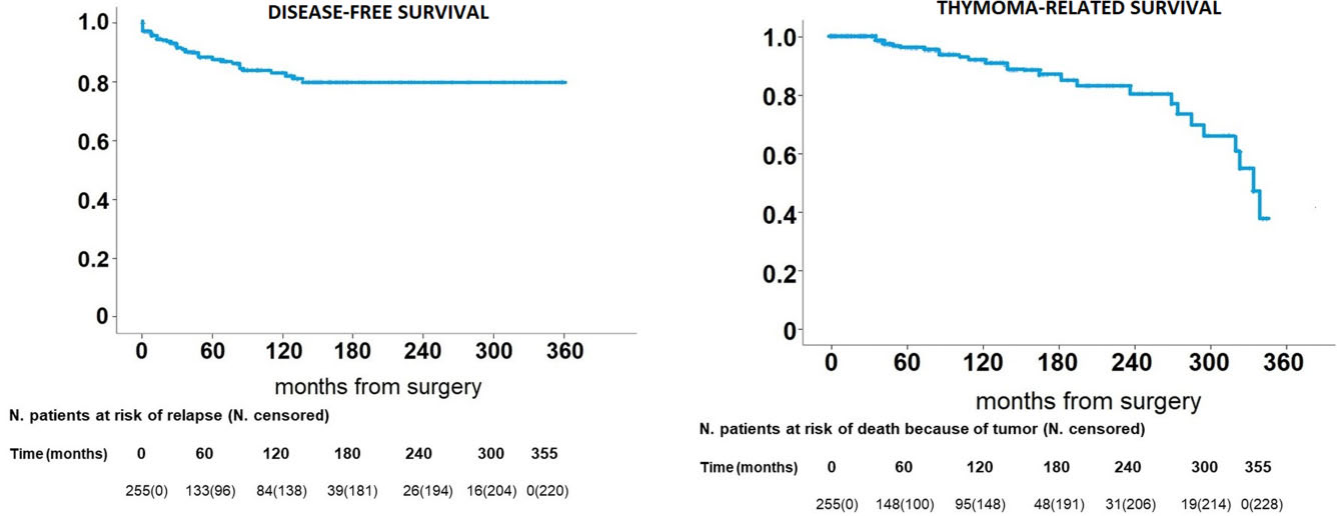
Disease-free survival and thymoma-related survival of all population.

Operations were performed through 65 minimally invasive (2 uniportal, 1 subxiphoid, 57 triportal and 5 robotics) and 190 open (108 sternotomies and 82 thoracotomies) approaches and as expected thymomectomies had been more frequently carried out through minimally invasive surgery (*P* < 0.001). Recurrence was more frequent after a complete thymectomy (24 cases) than after a thymomectomy (11 cases) (*P* = 0.015). This result was expected since the follow-up of the former procedure was longer than the follow-up of the latter (153.6 ± 107.8 vs 64.1 ± 51.5 months) (*P* < 0.001). There was no evidence of a difference in the number of deaths and deaths related to thymoma between the 2 groups. Disease-free and thymoma-related survivals are shown in Fig. 2 and reported a 5-year rate of 87.7% and 96.0% and a 10-year rate of 82.2% and 91.9%, respectively. Finally, 6 (4.8%) patients developed a post-thymectomy myasthenia gravis and 3 (2.3%) patients developed a post-thymomectomy myasthenia gravis after a mean period of 34.4 ± 18.5 months. Thus, the frequency was more elevated after thymectomy but the mean follow-up is too short to realize the true incidence of late manifest myasthenia. Another 3 developed other autoimmune diseases: hepatitis (*n* = 1), polymyositis (*n* = 1) and red cell aplasia (*n* = 1) ranging from 6 to 18 months from surgery always after thymomectomy. Interestingly, this interval was significantly shorter (*P* = 0.025).

### Propensity score-matching analysis

Propensity score extrapolated a group of 176 (88 thymectomies and 88 thymomectomies) patients. After matching the 2 groups of patients were statistically homogeneous for age, gender, tumour diameter, approach, WHO histology and pathological staging (Table 1).

The mean follow-up time of the thymomectomy group was significantly shorter than that of the complete thymectomy group (59.6 ± 54.8 vs 141.7 ± 103.8 months) (*P* < 0.001). Disease-free interval presented the same behaviour, being shorter in the former group (57.9 ± 54.6 vs 122.9 ± 104.8 months) (*P* < 0.001). These results are expected since thymomectomy was introduced later than complete thymectomy (2010 versus 1986). Standardized differences in pre- and post-matching are reported in Table 1.

We suffered 2 deaths within 30 days off the procedure in both groups. The postoperative major morbidity rate at 30 days after surgery was 3% (3/88) vs 7% (6/88) and it was significantly higher in the thymectomy group (*P* = 0.04). Postoperative major morbidities were low respiratory tract infection (*n* = 2 vs *n* = 2), haemothorax (*n* = 2 vs *n* = 1), wound infection (*n* = 1 vs none) and atrial fibrillation (*n* = 1 vs none). In the case of extended resection for limited disease, we have observed only few cases of temporary nerve palsy recovered in a short time period without impact on major morbidity.

### Survival analysis

After propensity score matching, we reported a total of 35 deaths, 22 (25.0%) occurred in the group undergoing thymomectomy and 13 (14.8%) in the thymectomy group. Deaths directly or indirectly due to thymoma evolution were distributed without statistical significance (3 vs 8, *P* = 0.12). Six (6.8%) patients recurred in the thymomectomy group and 16 in the thymectomy (18.2%) group (*P* = 0.023). Interestingly, local recurrences were not more frequent among patients undergoing thymomectomy (3, 50.0% vs 4, 25.0%). This result is in contrast with the general opinion that a limited resection would be associated with a greater incidence of local recurrence.

**Figure 3:**
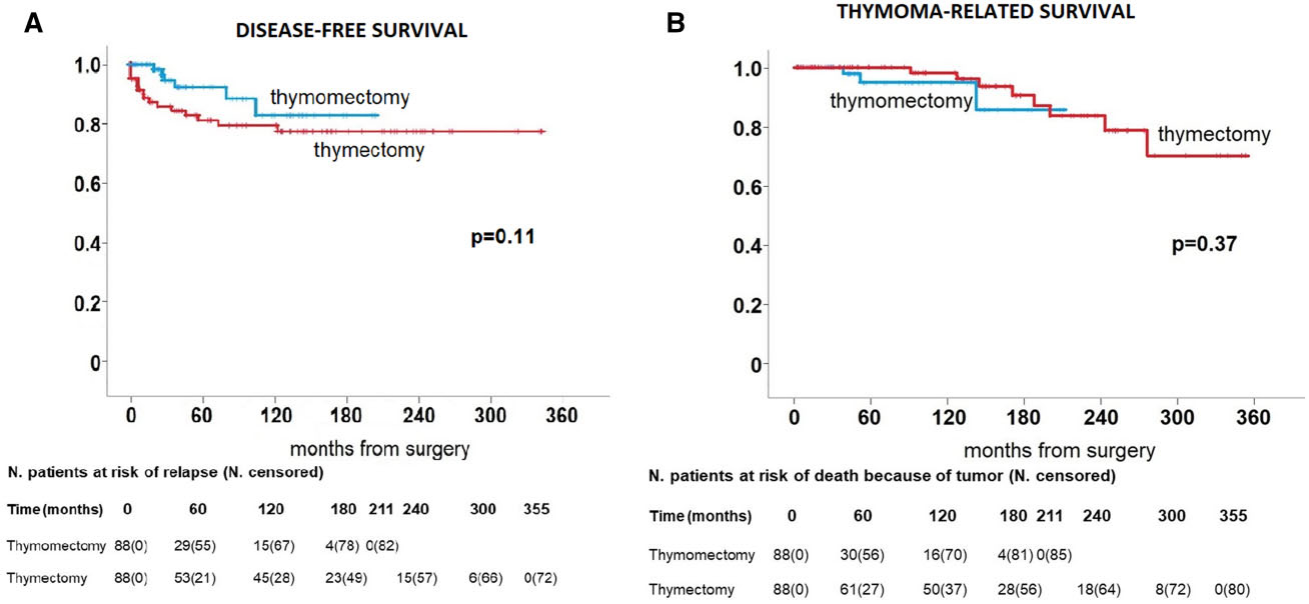
(**a**) Disease-free survival in the 2 group of patients. (**b**) Thymoma-related survival in the 2 groups of patients.

Thymoma-related survival did not disclose significant differences between groups (*P* = 0.37) (Fig. 3a) with 5- and 10-year survival rates of 100.0% and 98.2% in the thymectomy group vs 95.1% and 95.1% in the thymomectomy group, respectively.

Similarly, there was no significant difference in disease-free survival between the 2 groups (*P* = 0.11) (Fig. 3b) showing a 5-year disease-free survival rate of 82.4% in the thymectomy group and 92.8% in the thymomectomy group, respectively.

Finally, 1 patient developed new MG after 36 months from the thymectomy and 2 after 7 and 36 months from thymomectomy, respectively. None of the 2 procedures was significantly associated with the development of the disease.

Multivariable Cox regression analysis (Table 2) showed that B2–B3 histology (*P* < 0.001), presence of residual disease (*P* < 0.001) and need of adjuvant chemotherapy (*P* < 0.001) were the only predictors of shorter disease-free survival. Whereas neither univariable nor multivariable Cox regression showed evidence to confirm that disease-free and thymoma-related survivals were influenced by resection extent.

**Table 2:** Results of univariable and multivariable analyses for disease-free survival and thymoma-related survival

	Disease-free survival			Thymoma-related survival	
	Univariable	Multivariable		Univariable	Multivariable
	*P*-Value	HR (95% CI)	*P*-Value	*P*-Value	HR (95% CI)
Age (<56 versus ≥56) years	0.90	–	–	0.98	–
Gender (M versus F)	0.75	–	–	0.14	–
Charlson index (0–2 versus 3–7)	0.35	–	–	0.29	
WHO histology (A, AB, B1 versus B2, B3)	<0.001	5.5 (2.0–15.0)	0.001	0.16	–
Masaoka stage (I versus II, III)	0.010	–	0.93	0.99	–
Tumour size (<63 versus ≥63) mm	0.024	–	0.16	0.22	–
Extent of resection (thymomectomy versus thymectomy)	0.12	–	–	0.38	–
Surgical approach (minimal versus open)	0.69	–	–	0.69	–
Residual tumour (absent versus present)	<0.001	7.5 (2.7–21.0)	<0.001	0.95	–
Chemotherapy (none versus adjuvant)	<0.001	1.6 (1.0–2.6)	0.048	0.93	–
Radiotherapy (none versus adjuvant)	0.18	–	–	0.36	–

F: female; CI: confidence interval; HR: hazard ratio; M: male; WHO: World Health Organization.

## DISCUSSION

Despite the numerous attempts of defining the various extent of resection, some doubts still persist [1, 3–5, 10, 15–19]. To avoid confusion or misunderstanding, we tried to synthetize all the definitions proposed in the literature about resection of thymoma with a particular caveat for the limits of conservative surgery.

Actually, complete thymectomy [1, 3] and thymothymectomy [10], defined as complete removal of the tumour mass *en bloc* with the entire thymus gland, appear to be the same procedure and represent our technique when we consider extended operations. Zielinski [19] introduced the maximal thymectomy providing the resection of tumour, thymus gland and mediastinal fat tissue including a neck dissection with sternal lifting performing with a mini-invasive approach. Korean Association of Research of Thymus and others introduced the definition of limited thymectomy considering the removal of tumour with surrounding thymus and fat tissue sparing the healthy thymus gland [4, 5].

Conversely, thymomectomy significantly differs: it represents a conservatory option reserved in early-stage thymoma without myasthenia gravis [4, 16]. In our series, thymomectomy consists of removal of the sole tumour and its capsule together with the adjacent and indissociable thymus gland without the residual thymic and fatty tissue and upper poles when it possible, thus representing the most conservative approach described so far [2, 17, 18].

Once having classified the different typology of the resections, one should deal with the effectiveness of a limited resection. Two main concerns against minimal resection are the increased probability of local recurrence and the occasional yet described postoperative newly onset of a formerly silent myasthenia gravis [15] or other autoimmune diseases. Indeed residual neoplastic foci or ectopic thymic tissue within the mediastinal fat can trigger the onset of these conditions. Minimally invasive approaches are lacunose in visualizing contralateral anatomical structures making it difficult and dangerous the clearance of all potential sites of neoplastic recurrence or of new-onset myasthenia. These end points can be effectively demonstrated only with a very long follow-up.

Evidence on the efficacy of more conservative resection in early-stage thymoma began to appear from 2010 with Onuki *et al.* [4]. In this retrospective study involving only 79 patients, no difference regarding 10-year disease-free and overall survival was found between complete and limited thymectomy nor in the incidence of postoperative myasthenia. However, patients with preoperative myasthenia were included and complete thymectomy was performed if the disease was present. In addition, complete thymectomy was performed solely through median sternotomy while limited thymectomy could also be achieved with other approaches (e.g. anterolateral or parasternal thoracotomy) and VATS was not used. Narm *et al.* performed a multi-institutional propensity-matched study, enrolling 762 patients with stage I and II non-myasthenic thymoma who underwent complete or limited thymectomy either by VATS or sternotomy. Again, no difference was found in terms of 10-year overall and disease-free survival after matching [5]. Tseng *et al.* performed, in 2013, an even more conservative resection, known as thymomectomy, on 42 patients and compared 5-year disease-free survival with another group of 53 patients who underwent complete thymectomy. All of the patients had stage I or II thymoma without myasthenia, and those with neoadjuvant chemotherapy were excluded. No difference was found between the 2 groups; however, the median follow-up is too short for such an indolent neoplasm and sample size small [16]. In 2016, Nakagawa *et al.* retrospectively analysed medical reports from 32 hospitals and matched, using propensity score analysis, a total of 552 patients with stage I and II non-myasthenic thymoma who underwent either complete thymectomy or thymomectomy [8]. They found a comparable 5-year overall survival between the 2 surgical approaches; however, follow-up time is limited and groups were not homogeneous since VATS was solely excluded from complete thymectomy group.

The results achieved by the present propensity score-matching study confirm those findings. As far as disease-free survival, which is the most important end point in a slow-growing tumour, we observed a slightly better yet unexpected survival in the thymomectomy group. Namely, if local recurrences were similar between groups, distant relapses were by far more frequent after thymectomy. We can explain this result because the patients who underwent thymectomy were followed for a significantly longer period (*P* < 0.001).

The ESTS Thymic working Group Study recently reported that patients with early-stage thymoma without myasthenia gravis performed with complete thymectomy have significantly better oncological outcome compared to patients who received conservative surgery [10]; however, in this series, the number of simple thymomectomy was relatively inferior compared to the number of extended operations (1:3).

Another interesting point is the onset of postoperative before silent myasthenia gravis. In our raw series, this event was mostly related after thymectomy; however, these data could have been influenced by the longer follow-up period. On the contrary, the patients who postoperatively developed other autoimmune diseases always underwent thymomectomy within a significantly shorter time period. However, our data are comparable with results of a Japanese study who analysed the reports of 115 institutes of the Japanese Association for Chest Surgery (1093 patients) and they found some rare cases of myasthenia gravis and other autoimmune diseases after thymic surgery [20]. They concluded that the onset of myasthenia gravis after total thymoma removal occurred in 1–3% of thymoma patients without myasthenia gravis and the resection of the thymus gland does not prevent the onset of the postoperative myasthenia gravis; these results are confirmed from other older study [21, 22]. However, this topic skips from our preliminary objective and will deserve further as well as more focused speculations.

### Limitations

We are perfectly aware that this study presents several limitations, mainly those related to the different types of staging used along the wide time span (Masaoka versus TNM). Unfortunately, this as a *posteriori* criterion and data for clinical staging are not homogeneous and unreliable due to the wide time period of data collection. Moreover, the study involved a limited number of consecutive cases at 2 institutions and, because of its retrospective nature, the treatment was not carried out according to the protocol and sometimes the extent of resection was decided by individual surgeons, potentially introducing selection bias. So these results should be interpreted with caution and longer follow-up period, especially for the extended thymomectomy group, will be required.

We must also denounce a significant difference in the 2 groups about the greater use of adjuvant chemotherapy after thymectomy. This finding was unexpected and might evoke some perplexities about the correct pairing between groups. As a matter of fact, in doing propensity score matching, we excluded all patients with preoperative knowledge of advanced disease and this discrepancy was completely unpredictable before surgery. Moreover, no other preoperative available data (Charlson comorbidity index, tumour size and surgical approach) could have influenced the entity of the resection. We could speculate that the use of adjuvant chemotherapy was more liberal in our centres in the last century when majority of the thymectomy were done.

## CONCLUSION

We can conclude that according to our propensity score-matching analysis, extended thymomectomy appears oncologically adequate as thymectomy in surgical therapy for non-myasthenic thymoma. Patients undergoing extended thymomectomy achieved disease-free and thymoma-related survivals comparable to those after complete thymectomy whatever the surgical approach.

**Conflict of interest:** none declared.

## Author contributions

**Emanuele voulaz:** Visualization; Writing—original draft. **Gianluca Perroni:** Data curation. **Alexandro Patirelis:** Data curation. **Anna Russo:** Resources. **Giuseppe Mangiameli:** Data curation. **Marco Alloisio:** Supervision. **Vincenzo Ambrogi:** Conceptualization; Formal analysis; Writing—original draft.

## Reviewer information

Interactive CardioVascular and Thoracic Surgery thanks Tevfik Kaplan, Larry R Kaiser, Haruhisa Matsuguma and the other, anonymous reviewer(s) for their contribution to the peer review process of this article.

## Notes

Presented at the 29th European Conference on General Thoracic Surgery, 20–22 June 2021 (VIRTUAL).

## ABBREVIATIONS

VATS: Video-assisted thoracic surgery
WHO: World Health Organization
